# A Systematic Review and Meta-Analysis Protocol of Chemoablation vs. Transurethral Resection of Bladder Tumor in Patients With Non-Muscle-Invasive Bladder Cancer

**DOI:** 10.3389/fsurg.2021.753547

**Published:** 2021-11-15

**Authors:** Xu Shi, Dechao Feng, Wuran Wei

**Affiliations:** Department of Urology, Institute of Urology, West China Hospital, Sichuan University, Chengdu, China

**Keywords:** chemoablation, chemoresection, instillation, non-muscle-invasive bladder cancer (NMIBC), transurethral resection of bladder tumor (TURBT), meta-analysis

## Abstract

**Background:** Bladder cancer is the second-ranked tumor of the genitourinary system. Transurethral resection of bladder tumor (TURBT) is currently the most important diagnosis and treatment method for non-muscular invasive bladder cancer (NMIBC). However, due to its high recurrence and progression rate, as well as high cost and inapplicability to some patients, intravesical chemoablation as an alternative to TURBT may be promising for NMIBC patients. However, there are very little data comparing its effectiveness, safety, best effective drug type, dosage selection, and cost with TURBT at present, which deserves further evaluation. The present study was designed in order to discuss which treatment is superior to another between chemoablation and TURBT in patients with NMIBC.

**Methods and Analysis:** Databases including PubMed, MEDLINE, EMBASE, and Cochrane Library databases, as well as Chinese databases including CNKI (China national knowledge infrastructure), Wan Fang database, and Chinese Clinical Trial Registry, from August 1994 to the time when the official submission of this review was published was included in this review and screened by two reviewers (XS and DCF) independently. There were no language limitations. The study was conducted according to Preferred Reporting Items for Systematic Reviews and Meta-Analyses (PRISMA). Data was analyzed using RevMan and Stata software. The primary aims were the clinical effectiveness, including response rate, complete response OS, CSM, recurrence rate, time to recurrent, progression rate, and time to progression, among others. The secondary aims mainly included safety and tolerability, including costs, operation time, hospital stay, bleeding volume, and complications, among others.

**Study Registration:** This study is registered as PROSPERO CRD42021271124.

## Introduction

The incidence of bladder cancer ranks 9th among malignant tumors in the world, 7th for men and 10th for women, and 13th for malignant tumors in mortality (1). Among them, urothelial cancer is the most common, accounting for more than 90% of bladder cancer, of which non-muscular invasive bladder cancer (NMIBC) accounts for 70% (2). NMIBC can then be classified as low, intermediate, or high risk, depending on the presence of characteristics (such as grade, stage, tumor size, number of tumors, presence of carcinoma *in situ*, and prior recurrence rate) that increase the likelihood of progression to muscle-invasive bladder cancer (MIBC) (3). A 0.8–6% risk of progression to muscle-invasive disease or bladder cancer death within 5 years and a relatively high rate of local recurrence, 46–62%, were observed in NMIBC (3–5). Regarding the high recurrence rate among even low-risk tumors, patients are often forced to receive repeated TURBT or other treatments, which brings high costs, lower living quality, and management costs. In addition, although TURBT is the standard procedure for the treatment of NMIBC, there are also some challenging situations, such as a TURBT with an NMIBC located in an inaccessible position, a large prostate or urethral stricture precluding the resectoscope introduction or an extensive low-grade Ta lesion that cannot be endoscopically resected. Also, a large amount of evidence shows that in >90% of cases, the recurrence of Ta low-grade tumors after TURBT is low-grade, and the risk of progression is negligible (6). Based on this evidence, safe alternative procedures for TUR have been proposed. However, there is no persuasive evidence to guide NMIBC handling outside of TURBT at present. Chemoablation may be a viable treatment for NMIBC.

Since 1972, a large number of studies have shown that intravesical treatment with doxorubicin (adriamycin) is effective against carcinoma *in situ* and multiple papillary tumors (7). In 1982, Koontz et al. first reported the ablative effect of intravesical therapy on the incompletely resected visible tumor (single or multiple) with a complete response (CR) of 47% through thiotepa (8). In 1994, researchers discovered that only intravesical treatment (IVU) can ablate isolated marked lesions left in the bladder after TURBT, and then began the exploration of the possibility of chemical ablation of NMIBC (9). Commonly used drugs for chemoablation include mitomycin C (MMC), epirubicin, gemcitabine, and bacillus Calmette-Guérin (BCG), to name a few.

Reviews of chemoablation (including >1,200 patients with varying risk and different chemotherapy regimens) suggest the complete response rate is 50%, with the therapeutic effect sustained for at least 2 years, suggesting that chemoablation may be a viable treatment for low-risk NMIBC (10, 11). There are currently no studies comparing different ablation drugs. To date, MMC is still one of the most widely used drugs for ablation and prevention of NMIBC recurrence. The latest randomized feasibility trial in 2020 showed that chemoablation with MMC against recurrent low-risk NMIBC reached a complete response of 37% at 3 months compared with 80% complete response in the surgical group, but the MMC group shows a higher 12-month recurrence-free proportion among both with or without residual disease at 3 months, which suggested that MMC may be feasible to prevent the recurrence of NMIBC and a more intense (three times per week) and more extended period (2 weeks) chemoablation with MMC might be more effective (12). Other studies using MMC have shown a higher CR rate: Colombo et al. reported a CR rate of 70.4%, and the rest were between 50 and 57% (6, 10, 13). It is worth noting that Lindgren et al. found that Complete tumor response was seen in 57% among patients treated with short-term, intensive chemoresection with MMC and with fewer adverse events compared with TURBT + adjuvant instillation in patients with recurrent NMIBC (13). The heterogeneity of these studies may come from the condition of the patient and the operation skills of the doctor. The CR rate of NMIBC patients obtained using epirubicin is between 56 and 67% (10, 14). While Gemcitabine is about 30% (15). It is worth noting that although there have been studies showing that the 5-year recurrence-free rate for the epirubicin plus Ara-C Instillation group (58.5%) was higher than that of the TURBT-only group (38.6%) (16). But the latest large RCT prompts Immediate post-TURBT epirubicin installation is ineffective for cancer recurrence or progression in intermediate and high-risk NMIBC (17). In general, chemotherapeutic agents are used in low-grade disease and BCG for high-grade disease including carcinoma *in situ* (CIS) (18). The study of Akaza reported a CR in 66.4% of the papillary tumors and a CR of 84% for CIS (19).

After reviewing the existing guidelines and literature, we will decide whether to recommend chemoablation as an alternative treatment to TURBT and to determine the best ablation plan and evaluate the patients who are most suitable for ablation and can thus avoid surgery.

## Methods

### Patients and Public Involvement

Patients and the public were not involved in this study.

### Eligible Criteria for Study Selection

#### Types of Studies

Our research only included RCTs. Only RCTs that showed an effect estimate for the comparison of the effectiveness or safety between chemoablation and TURBT, or an effect estimate for the comparison of the effectiveness or safety of different chemoablation drugs, or those comparing patients with different baselines will be included. The language of the article was not restricted.

#### Types of Participants

Human patients diagnosed with NMIBC (Ta, T1, and TIS), based on 2017 UICC TNM staging, was included. Diagnosis relied on the results of cystoscopy and pathological biopsy or diagnostic TUR and pathological examination, regardless of initial diagnosis or recurrence. Additionally, urothelial carcinoma of the upper urinary tract was excluded by radiology.

#### Types of Interventions

Interventions included the implementation of chemoablation of any kind of drugs and doses. The control group implemented TURBT. The two groups could have other treatment measures but must be consistent.

### Types of Outcome Measures

#### Primary Outcomes

The primary aim was the clinical effectiveness, including response rate, complete response OS, CSM, recurrence rate, time to recurrent, progression rate, and time to progression, among others.

#### Secondary Outcomes

The secondary outcomes included safety and tolerability, such as costs, operation time, hospital stay, bleeding volume, and complications, among others.

#### Searching Methods for the Identification of Studies

Databases including PubMed, MEDLINE, EMBASE, and Cochrane Library databases, as well as Chinese databases including CNKI, Wan Fang database, and Chinese Clinical Trial Registry from August 1994 to the time when the official submission of this review was published was included in this review and screened by two reviewers (XS and DCF) independently with no language limitations. The above-mentioned electronic databases was searched for all possible combinations of these terms, mainly in the titles, keywords, and abstracts: “chemoablation,” “chemoresection,” “instillation,” “TURBT,” “transurethral resection of bladder tumor,” “bladder tumor,” “NMIBC,” and “non-muscle-invasive bladder cancer.” In addition, we manually screened the reference lists of the retrieved articles and relevant articles for further material for inclusion.

### Data Collection

#### Selection of Studies

**Figure 1:**
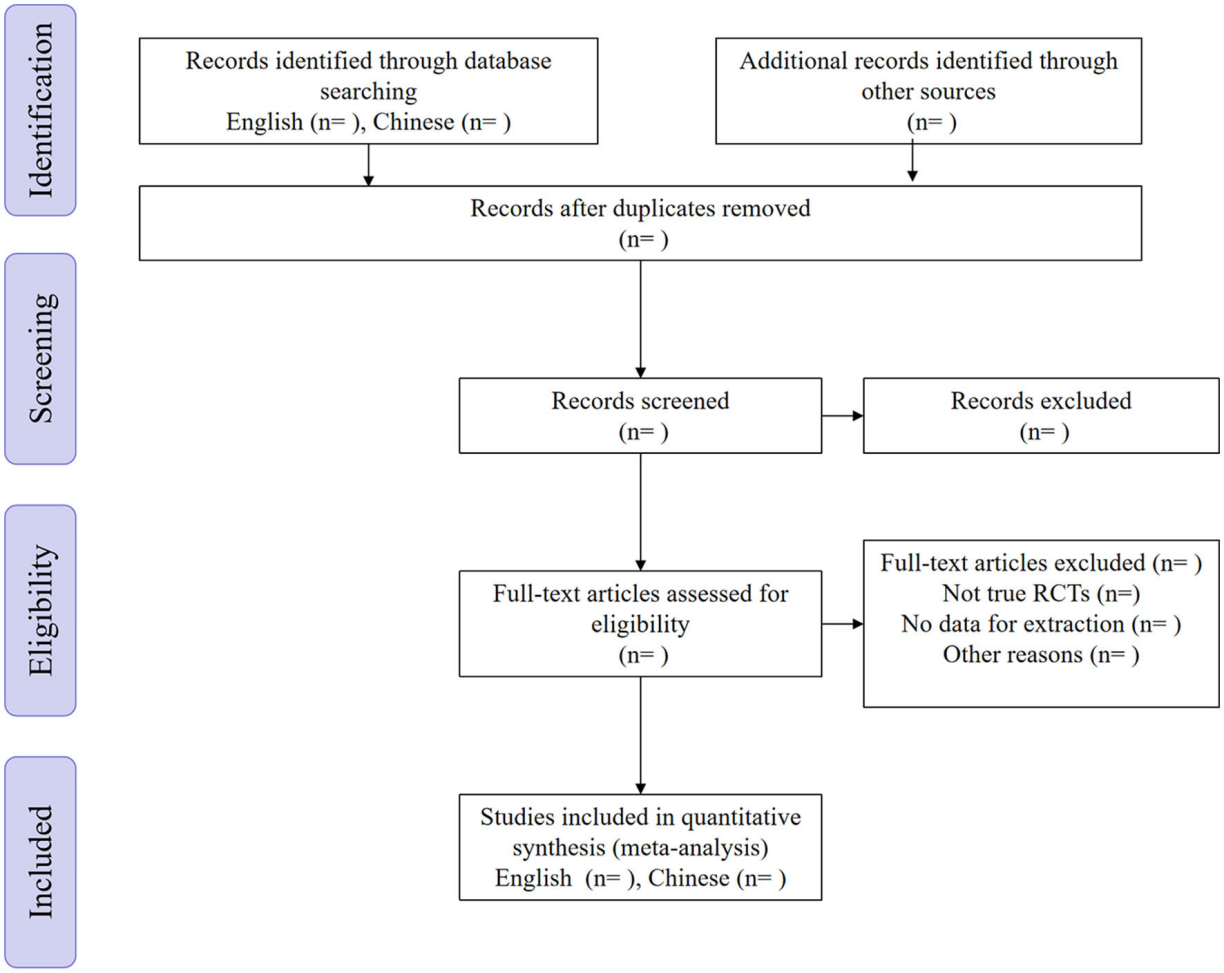
Flow diagram of the study selection process. RCT, randomized controlled trial.

All the exported documents were first reviewed independently by the two authors (XS and DCF) according to title and abstract and made a preliminary evaluation. The selected documents were then re-evaluated by the original text. Finally, we searched for cited documents and use the “related articles” function. Articles screened by the two authors were checked for consistency. Any objections were decided by another author (WRW) after discussion (Figure 1).

### Data Extraction and Management

The two authors (XS and DCF) had separately extracted the following content: author, publication year, follow-up time, and baseline indicators in each article (patient age, recurrence, tumor size, number, and grade, to name a few), intervention and control measures (types and dosage of drugs used in chemoablation, among others), outcome indicators (response rate, complete response OS, CSM, recurrence rate, time to recurrent, progression rate, and time to progression, to name a few), safety and tolerability indicators (costs, operation time, hospital stay, bleeding volume, and complications, among others). If there was a dispute, it was decided by the third author (WRW) after discussion. For primary studies where the original data was not mentioned in the original text and its Supplementary Material, we tried to contact the author to obtain the original data. If not successful, we imported the image into Engauge Digitizer 10.8 for approximate calculation.

### Assessment of Risk of Bias

We will use the Cochrane Collaboration tool for assessing the risk of bias (ROB) to evaluate the risk of bias for RCTs, which covered sequence generation, allocation concealment, blinding, incomplete outcome data, selective reporting, and other biases. Two authors (XS and DCF) had evaluated the bias of the literature independently and rate it as “high risk,” “low risk,” or “unclear risk.” For publication bias, we will use a funnel plot and Egger's test to display an electronic graph. If duplications were found, we selected those with the latest publication, the largest sample size, and the longest follow-up time. If there was a difference, it was determined by the third author (WRW).

## Data Analysis

### Data Synthesis

We used Review Manager version 5.3 (Nordic Cochrane Centre [Cochrane Collaboration], Copenhagen, Denmark) and Stata software, version 16.0 (Stata Corp., College Station, TX, USA) for data analysis. EndNote version 20 (Clarivate, Philadelphia, PA, USA) was be used for sorting references. We evaluate the baseline characteristics, type of intervention, the outcome indicators and safety, and tolerability indicators to combine the same type of statistics to estimate the effect with a relative risk (RR) or hazard ratio (HR) with 95% CI. We conducted a Der Simonian random-effects model for within-study and between-study variations. If the indicators were too heterogeneous or the indicators could be combined, a descriptive analysis will be performed.

### Assessment of Heterogeneity

We assessed the heterogeneity using the χ^2^-test and *I*^2^ statistics. RevMan was used for the heterogeneity test, selection effect index, and statistical model. We used the χ^2^-test for statistical heterogeneity analysis (inspection level a = 0.1) to estimate heterogeneity size. If the heterogeneity was not statistically significant (*I*^2^ <0.5 and *p* > 0.1), the fixed effects model was used for analysis. On the other hand, if the heterogeneity was statistically significant (0.5 < *I*^2^ or *p* < 0.01), the random-effects model was used. The relative risk (RR) or hazard ratio (HR) was used as the statistical quantity of the enumeration data, and each effect size was expressed by 95% CI. We will pool the results using a two-sided *P*-value of < 0.05 for each outcome.

### Subgroup Analysis and Sensitivity Analysis

If the necessary data were available, subgroup analyses was done for baseline characteristics (patient age, recurrence, tumor size, number, and grade, among others), the different drug type and dosage [e.g., mitomycin C (MMC), epirubicin, gemcitabine and *bacillus* Calmette-Guérin (BCG)], different types of the control group (TURBT with or without adjuvant therapy). We also showed the results of the sensitivity analysis to give a relatively robust outcome.

### Grading the Quality of Evidence

We used the Grading of Recommendations Assessment, Development, and Evaluation guidelines (GRADE) to grade the quality of included RCT studies.

## STRENGth And Limitations Of This Study

1. This meta-analysis will be the first analysis to compare chemoablation and TURBT for NMIBC patients.
2. Chemoablation has not yet been written into the primary management plan of NMIBC. This article will help patients who are not suitable for TURBT to provide another option.
3. The reliability of the research results depends on the authenticity and credibility of the original research data, of which we will also make an analysis.

## Data Availability Statement

The original contributions presented in the study are included in the article/Supplementary Materials, further inquiries can be directed to the corresponding author.

## Author Contributions

XS and DF: conception and design, provision of study materials or patients, collection and assembly of data, and data analysis and interpretation. WW: administrative support. All authors: manuscript writing and final approval of manuscript.

## Funding

The study was supported by the Department of Science and Technology of Sichuan Province (2020YFH0099). The funders had no role in study design, data collection or analysis, preparation of the manuscript, or the decision to publish.

## Conflict of Interest

The authors declare that the research was conducted in the absence of any commercial or financial relationships that could be construed as a potential conflict of interest.

## Publisher's Note

All claims expressed in this article are solely those of the authors and do not necessarily represent those of their affiliated organizations, or those of the publisher, the editors and the reviewers. Any product that may be evaluated in this article, or claim that may be made by its manufacturer, is not guaranteed or endorsed by the publisher.

## Abbreviations

TURBT: transurethral resection of bladder tumor
NMIBC: non-muscle-invasive bladder cancer
MIBC: muscle-invasive bladder cancer
MMC: mitomycin C
BCG: bacillus Calmette-Guérin.
