# Supplementary material for: A Systematic Review and Meta-Analysis Protocol of Chemoablation vs. Transurethral Resection of Bladder Tumor in Patients With Non-Muscle-Invasive Bladder Cancer
Source: Front Surg. 2021 Nov 15;8:753547. doi: 10.3389/fsurg.2021.753547 (PMC8634134; doi:10.3389/fsurg.2021.753547)
Supplement: Supplementary file 1 [file Data_Sheet_1.PDF]

Supplementary file: research strategy in PubMed.

((((((((((Urinary Bladder Neoplasm*) OR (Bladder Neoplasm*)) OR (Bladder Tumor*)) OR (Urinary Bladder Cancer*)) OR (Malignant Tumor of Urinary Bladder)) OR (Cancer of the Bladder)) OR (Bladder Cancer*)) OR (Cancer of Bladder)) OR (NMIBC)) OR (non-muscle-invasive bladder cancer)) AND ((TURBT) OR (transurethral resection of bladder tumor))) AND (((chemoablation*) OR (chemoresection*)) OR (instillation*))
